# Modulation of mitochondrial function by methylene blue and phosphorylated tau protein

**DOI:** 10.1192/j.eurpsy.2026.11617

**Published:** 2026-07-31

**Authors:** J. Hroudová, K. Škutová, S. Krasňan, Z. Fišar

**Affiliations:** 1 Department of Psychiatry; 2 Institute of Pharmacology, First Faculty of Medicine, Charles University and General University Hospital in Prague, Prague, Czech Republic

## Abstract

**Introduction:**

Regulation of mitochondrial function is a promising approach for the treatment of neurodegenerative diseases such as Alzheimer’s disease (AD). Methylthioninium chloride, also known as methylene blue (MB), is a well-known antioxidant that improves mitochondrial function and reduces neuroinflammation. Due to its ability to inhibit the aggregation of phosphorylated tau (P-tau), MB is a promising therapeutic agent in the treatment of neuropsychiatric diseases, including AD (Tucker *at al.* Mol Neurobiol 2018; 55 5137-5153).

**Objectives:**

The aim of our study is to investigate mitochondrial effects of MB and P-tau oligomers in isolated brain mitochondria.

**Methods:**

The *in vitro* effects of MB on mitochondrial function were measured using high-resolution respirometry with simultaneous fluorimetric determination of hydrogen peroxide (H_2_O_2_) or ATP production. The dose-dependent effects of MB were measured at concentrations ranging from 0.05 to 30 μmol/l, and the effects of P-tau were measured at concentrations of 30 and 60 nmol/l.

**Results:**

We found the following dose-dependent effects of MB: (1) Dose-dependent increases in mitochondrial oxygen consumption for complex I-linked (N-type) respiration and decreases for complex II-linked (S-type) respiration; (2) Increases in mitochondrial H₂O₂ production for N- and S-type respiration, reaching a maximum at 10 μM MB; and (3) Decreases in ATP production, especially for S-type respiration. MB most significantly affects mitochondrial respiration and H_2_O_2_ production in respiratory states 3 and 4. P-tau alone does not significantly affect respiration or H₂O₂ production (Fišar *et al.* Biomolecules 2025;15 495). However, P-tau potentiates the decrease in MB-induced S-type respiration. At 1 μmol/l MB, P-tau increases H_2_O_2_ production in N-type respiration and decreases H_2_O_2_ production in S-type respiration.

**Conclusions:**

The neuroprotective effects of MB appear to be associated with changes in the activity of complex IV of the respiratory chain rather than with its antioxidant effects. We confirmed that MB transfers electrons directly from complex I to cytochrome c and complex IV, thereby increasing complex IV activity as well as oxygen consumption. The increase in complex IV activity due to MB could have a beneficial effect in AD, where complex IV activity is typically reduced. However, the MB-induced increase in H₂O₂ production and decrease in ATP production suggest that MB may negatively affect mitochondrial function. The toxic effects of MB may be associated with the disruption of mitochondrial energy metabolism through direct damage to the inner mitochondrial membrane, uncoupling of oxidative phosphorylation, or inhibition of electron transport.

Supported by Ministry of Health of the Czech Republic, NU23-04-00032.

**Disclosure of Interest:**

None Declared

